# The distribution structure of medical and care resources based on regional characteristics throughout Japan in 2020

**DOI:** 10.1186/s12913-024-10699-5

**Published:** 2024-02-20

**Authors:** Takuya Kusunoki, Tohru Yoshikawa

**Affiliations:** Department of Architecture and Building Engineering, Graduate School of Urban Environmental Sciences, Tokyo Metropolitan University, Tokyo, 192-0397 Japan

**Keywords:** Ministry of health, Labour and welfare, Aging population, Secondary medical area, Hospital bed, Nursing home, Regional characteristics, Principal component analysis, Structural equation modeling, Multigroup analysis

## Abstract

**Background:**

Given Japan’s rapidly aging population, the Ministry of Health, Labour and Welfare's policy of reducing hospital beds and replacing medical care with nursing care requires the establishment of a coordinated system of medical and care services tailored to regional characteristics. To gain useful knowledge for the development of such a system, this study aimed to identify differences in the structure of the relationship between medical and care resources due to differences in regional characteristics.

**Methods:**

Initially, regional characteristics were used to group all 334 secondary medical areas (SMA) in Japan by principal component analysis. Subsequently, the related structure of the distribution of medical and care resources for each group were compared. For these　comparisons, first, the related structure of the distribution of medical and care resources nationwide was modeled using structural equation modeling. Secondly, multigroup analysis was conducted to investigate differences among the models across groups.

**Results:**

The nationwide SMAs were grouped largely based on urbanicity and middle-density regionality. The groups with high urbanicity and high middle-density regionality consisted of SMAs with a high and medium population density. By contrast, the low middle-density regionality group consisted of SMAs containing large cities with a high population density and depopulated areas with a low population density. The model of the related structure of the distribution of medical and care resources differed among these groups. In the non-urbanicity and middle-density regionality groups, nursing care abundance tended to increase acute care abundance. In addition, in all groups, nursing care abundance tended to increase long-term hospitalization care abundance and clinic care abundance (with beds).

**Conclusions:**

The key finding of this study was that the government’s objective of reducing hospital beds may not be achieved solely by expanding nursing homes. This is because many of the models did not show a tendency that higher nursing care abundance reduces the values of the factors which increase more hospital beds. This finding was particularly relevant in middle-density regionality groups. This finding suggests that the location of nursing homes should be monitored because of concerns about the oversupply of nursing homes and sprawl in those areas.

## Background

Japan, with the fastest aging rate in the world [1], needs to respond to the escalating demand for medical care. However, despite having the highest number of hospital beds per population worldwide, the medical care density per hospital bed is low, resulting in reduced medical productivity and efficiency [2]. In addition, the increase in the number of older adults living alone may make discharge from the hospital more difficult. As of October 2020, approximately 75% of hospitalized patients were 65 years of age or older, and the average duration of hospitalization was long [3]. In 2014, the Ministry of Health, Labour and Welfare (MHLW) responded to this issue by requesting each prefecture to establish a regional medical vision, indicating the direction of the medical delivery system, including the structural modification of hospital beds [4]. This structural modification entails the replacement of general hospital beds and long-term care hospital beds with four new categories: advanced acute phase, acute phase, convalescent phase, and chronic phase. The estimated number of hospital beds required in 2025 was based on this categorization for each secondary medical area (SMA). SMAs are regional units established to adjust beds for hospitals and clinics (medical facilities with 19 beds or fewer) [5]. SMAs comprise several municipalities. Nationally, advanced acute- and acute-phase hospital beds will decrease by 30%, convalescent-phase hospital beds will increase threefold, and chronic-phase hospital beds will decrease by 20%. The threefold increase in convalescent-phase hospital beds suggests the MHLW’s strong desire to discharge hospitalized patients to their homes and replace the medical care for patients who have a long duration of hospitalization with nursing care. The 20% decrease in chronic-phase hospital beds will be replaced by nursing homes and home care [6]. In addition, the MHLW is requiring municipalities, the insurers in the long-term care insurance system, to establish “community-based integrated care systems” tailored to regional characteristics by 2025 [7]. These “community-based integrated care systems” are designed to provide an integrated approach to care, medical treatment, housing, livelihood support, and care prevention. Based on these regional medical visions, the MHLW emphasizes the need for collaboration between medical and care services in the basic policy of the Seventh Medical Care Plan, which commenced in 2018 [8].

In summary, the MHLW and municipalities in Japan are collaborating to replace medical care with nursing care services by establishing a system of cooperation between medical and care services tailored to regional characteristics in preparation for the country’s increasingly super-aging population. Therefore, by comprehending the distribution of medical and care resources in recent years, valuable insights will be gained, which will aid in the development of future medical and care coordination systems.

### Previous research

This section provides a summary of recent research exploring the allocation of medical and care resources, which is the focal point of the present this study. First, we review previous investigations on a global scale. Second, we provide a detailed review of research conducted in Japan. In particular, we investigate studies that explore the distribution of medical and care resources individually, as well as their allocation in combination.

Numerous studies investigating the allocation of medical resources have been carried out on a global scale. International comparative studies have indicated that Japan boasts an equitable dispersion of nurses, and its healthcare system garners high levels of public satisfaction [9, 10]. By contrast, a comparison with the USA reveals that physicians in the USA are inclined to gravitate toward areas with higher income, unlike in Japan, where no such pattern is seen; these distinguishing features are attributed to the existence of universal public health insurance in Japan [11, 12]. In addition, China exhibits regional variability in nursing homes and disparities in the diversity of facilities around the aged [13, 14]. Nevertheless, these studies are confined to analyzing medical and care resources in isolation, and thus, the relationship between the two resources remains poorly understood.

The following two Japanese studies have explored the relationship between regional characteristics and the distribution of medical resources in recent years. The first study, conducted by Hara et al. [15], analyzed changes in physician distribution trends across SMAs nationwide from 2000 to 2014. The results indicated a decrease in physician supply in all regions except urban areas. The second study, by Seo et al. [16], analyzed regional differences in the performance of medical delivery systems and factors impacting medical costs in municipalities in Tokyo (urban areas) and Chiba Prefecture (suburban areas) in 2018. The results revealed that while the medical delivery system was well-developed in urban areas, it remained inadequate in suburban areas. These studies suggested that medical delivery systems differed due to regional characteristics. The following two Japanese studies examined the estimated number of hospital beds in the regional medical visions. The first study, conducted by Takizawa et al. [17], surveyed medical social workers working at medical institutions with convalescent-phase hospital beds in Aomori Prefecture in 2019. The results indicated that increasing the number of convalescent-phase hospital beds up to the number estimated based on the regional medical vision was challenging because of the underdeveloped treatment environment in community and home care. The second study, by Miyazawa [18], compared the estimated number of hospital beds based on regional medical visions with the real number in 2005, targeting SMAs nationwide. The results suggested that the regional imbalance in medical supply cannot be resolved. These studies indicated the discrepancy between regional medical visions and the actual situation. In summary, the four studies introduced so far indicate that the uneven distribution of medical resources is related to regional characteristics. Moreover, these studies suggest that it is difficult to change the distribution of medical resources in some regions, even with interventional policies such as regional medical visions, and that even if such changes are made, medical resources are still unevenly distributed in some regions.

The following three Japanese studies have explored the distribution of care resources and the current state of care delivery systems in recent years. The first study, conducted by Ikeda et al. [19], analyzed the relationship between the number of deaths at home and the distribution of medical and care resources in municipalities nationwide in 2014 and 2017. The second study, by Jin et al. [20], analyzed the factors contributing to regional differences in the cost of care, targeting long-term care insurance users and municipalities nationwide in 2016. The results showed that there were considerable regional differences in care costs, and that municipalities in urban areas with more “welfare facilities for older adults requiring long-term care” (one type of nursing home that provides end-of-life care) per population were strongly associated with higher care costs. The third study, by Nishino et al. [21], estimated the number of users of long-term care insurance facilities nationwide in 2010, comparing it with the MHLW target in 2025. That study also examined the applicability of the MHLW target for the provision of long-term care insurance facilities in 2025 for a specific city in Ishikawa Prefecture. The results suggested that it is difficult to transition from nursing home care to home care when the older adults receiving care are living in single-person households. In summary, these three studies suggest the presence of regional differences in the distribution of care resources and care delivery systems. Moreover, these studies suggest that the policy of providing home medical care and home care through “community-based integrated care systems” will be promoted nationwide, but the distribution of care resources and the care provision system differ from region to region, suggesting that the promotion is difficult depending on regional characteristics.

The following two Japanese studies addressed variables related to regional characteristics and medical and care resources in an integrated manner. The first study, conducted by Ishikawa et al. [22], identified the characteristics of SMAs nationwide from 2008 to 2011, utilizing principal component analysis (PCA) with variables pertaining to regional characteristics and medical and care resources. The findings revealed that medical care adequacy was deficient in urban and remote areas. The second study, conducted by Kusunoki et al. [23], analyzed the interrelation between regional characteristics and medical and care resources using factor analysis and structural equation modeling (SEM) for SMAs nationwide in 2015. The results indicated that suburban areas with a higher concentration of older individuals tended to have better medical care adequacy, whereas urban areas tended to have higher acute care adequacy via universities with medical schools. Although these two studies provide some perspective on the structure of the relationship between regional characteristics and medical and care resources, both methods used integrated information on regional characteristics and the allocation of care resources. Consequently, the difference in the structure of the relationship between medical and care resources due to differences in regional characteristics remains unclear.

### Research gap and purpose of this study

The summary of the studies discussed in the preceding section indicates that the associated structure of the distribution of medical and care resources worldwide has not been thoroughly elucidated. Even in Japan, where collaboration between medical and care services is encouraged, many studies separate medical and long-term care resources or investigate limited target regions. In addition, in studies using multivariate analyses such as PCA or SEM, the difference in the structure of the relationship between medical and care resources due to differences in regional characteristics remains unclear. This is due to the limitations of the analysis methods, which have not been able to clarify the complex structure of relationships among variables. Therefore, the present study attempts to group all the SMAs of Japan using regional characteristics, and to compare the associated structure of the distribution of medical and care resources in each group so as to clarify the complex associated structure of variables. This study also aims to fill the existing research gap in which the difference in the structure of the relationship between medical and care resources due to differences in regional characteristics is unclear, thereby contributing to the future coordination of medical and care services.

## Methods

### Study design

This study was divided into three phases. Initially, in the first phase, PCA was applied to group all SMAs throughout Japan according to regional characteristics. Subsequently, we compared the associated structure of the distribution of medical and care resources for each group obtained in the first phase, the comparison consisted of the second and third phases as follows. In the second phase, SEM was conducted to build the associated structure of the distribution of medical and care resources in all of Japan. This is because a comparison of groups requires a model for the integrated groups. Finally, in the third phase, multigroup analysis was conducted to compare the models, where the model obtained in the second phase was applied to the groups obtained in the first phase.

By dividing the methodology into these three phases, the first phase deals with only variables of regional characteristics, while the second phase deals with only variables of the distribution of medical and care resources. The third phase integrates these results. Therefore, this study attempts to resolve the limitation in previous studies, i.e., the inability to clarify the complex associated structure among variables caused by its analysis methods that treated variables in an integrated manner. Thus, we expect our findings to fill the above-mentioned research gap.

In the following sections, we first define the target regions to be analyzed, and then provide a detailed description of the methodology of these three phases.

### Target regions

Japan’s medical administrative areas consist of, primary medical areas, tertiary medical areas, and SMAs. Primary medical areas are set up in units of municipalities and provide short-term hospitalization and outpatient visits. Tertiary medical areas are generally established on a prefectural basis and provide advanced acute-phase and specialized medical care. SMAs, which are located between these areas, consist of several municipalities, and are defined by each prefecture based on the ability to provide medical care. Additionally, the MHLW requires welfare areas for older adults (areas determining the expected volume of long-term care insurance service provision) to coincide with SMAs [24]. Whereas previous studies have primarily focused on analyzing the distribution of care resources at the municipal level, the target regions in this study are 334 nationwide SMAs, considering the distribution of medical and care resources simultaneously. The summary statistics of the populations and areas of the SMAs are presented in Table 1.

**Table 1:** Summary statistics of the populations and areas of the SMAs

Variable	Mean^†^	SD^†^	MIN^†^	Q1^†^	Q2^†^	Q3^†^	MAX^†^	Source
Population	377,325	458,306	19,122	98,405	216,477	473,336	3,777,491	[25]
Area (km2)	1111	1102	64	433	852	1404	10,828	

^†^The abbreviations Mean, SD, MIN, Q1, Q2, Q3, MAX are as follows

*Mean* Mean Value, *SD* Standard Deviation, *MIN* Minimum Value, *Q1* First Quartile, *Q2* Second Quartile, *Q3* Third Quartile, *MAX* Maximum Value

### First phase: PCA for grouping all of Japan according to regional characteristics

The regional characteristics used in the first phase consisted of 28 variables selected in reference to the study by Miyake et al. [26], which classified SMAs based on various regional characteristics. These variables are presented in Table 2 [25, 27–31] and the summary statistics are presented in Table 3. In addition, the reasons for the selection of each variable are presented in Table 4. The variables are classified into the following four categories: population, which indicates the age composition and population size; household category which indicates the composition of households; industry and work, which indicates the industrial composition, mode of transportation, and economic scale, and land utilization, which indicates the land use status and spatial characteristics. As most of these variables are available for the year 2020, it was used as the base year for the present study.

**Table 2:** The 28 variables related to regional characteristics

Category	Variable	Calculation method	Source
Population	Proportion of population aged under 15 years	Population aged under 15 years / population	[25]
	Proportion of population aged 65 years and over	Population aged 65 years and over / population	
	Proportion of population change	Population change over 5 years / population 5 years ago	
	Population density	Population / land area (km^2^)	
	Proportion of population in densely inhabited districts	Population in densely inhabited districts^†1^ / population	
	Proportion of daytime to nighttime population	Daytime population / 100 nighttime population	
	Proportion of internal migrants from other municipalities	Number of internal migrants from other municipalities / population	[27]
Household	Persons per household	Population / number of households	[25]
	Proportion of single-person households (excluding households aged 65 years and over)	Number of single-person households (excluding households aged 65 years and over) / number of households	
	Proportion of single-person households aged 65 years and over	Number of single-person households aged 65 years and over / number of households	
	Proportion of nuclear households	Number of nuclear households / number of households	
	Proportion of double-income households	Number of double-income households / number of households	
	Proportion of households living in a detached house	Number of households living in a detached house / number of households	
	Proportion of households living in government-owned rental housing	Number of households living in a government-owned (e.g., government management, urban renaissance agency, government corporation) rental housing / number of households	
Industry & Work	Proportion of workers in primary industry	Number of workers in primary industry / population	[25]
	Proportion of workers in secondary industry	Number of workers in secondary industry / population	
	Proportion of workers in tertiary industry	Number of workers in tertiary industry / population	
	Proportion of working population	Number of working population / population	
	Proportion of commuters using rail	Number of commuters using rail / population	
	Proportion of commuters using their own vehicle	Number of commuters using their own vehicle / population	
	Annual retail merchandise sales per capita	Annual retail merchandise sales^†2^ (JPY) / population	[28]
	Municipal residence tax per capita	Municipal residence tax^†3^ (JPY) / population	[29]
Land utilization	Proportion of densely inhabited district areas	Densely inhabited district areas / total area	[27]
	Proportion of land area used for building	Land area used for building^†4^ / total area	[30]
	Proportion of forest area	Forest area^†5^ / total area	
	Proportion of land area used for rice paddies and agriculture	Land area used for rice paddies and agriculture^†6^ / total area	
	Proportion of depopulated area	Depopulated area^†7, †8^ / total area	[31]
	Number of pre-merger municipalities	Number of previous municipalities that merged with municipalities^†9^ in the 2000 transition	[25]

^†1^The “densely inhabited districts” are areas designated by the “population Census” [25], where adjacent basic unit areas have a population density of 4000 persons or more /km2, and the population of these adjacent areas is 5000 persons or more

^†2^The “annual retail merchandise sales” data were obtained from the “Economic Census” data for 2016 [28] because data for 2020 were not available. To match the age of the population, the denominator used was the “Population Census” data for 2015 [25]

^†3^The “municipal residence tax” is a Japanese tax levied on individuals based on their income in the previous year, which is paid to the municipality in which they reside

^†4^The “land area used for building” was estimated from the tertiary land use mesh data using GIS. Data for 2020 were not available, so data for 2016 were used

^†5^The “forest area” was estimated from the tertiary land use mesh data using GIS. Data for 2020 were not available, so data for 2016 were used

^†6^The “land area used for rice paddies and agriculture” was estimated from the tertiary land use mesh data using GIS. Data for 2020 were not available, so data for 2016 were used

^†7^The “depopulated area” is defined in Japanese law, and is defined as a depopulated area or a partially depopulated area, based on the region’s rate of population decline and financial strength

^†8^The “depopulated area” data for 2020 were not available, so data for 2022 were used. The denominator used was the “Population Census” data for 2020 [25]

^†9^The “number of previous municipalities that merged with municipalities” was independently calculated by checking the “Population Census” data for 2020 [25]

**Table 3:** Summary statistics of the 28 variables related to regional characteristics

Category	Variable	Mean^†^	SD^†^	MIN^†^	Q1^†^	Q2^†^	Q3^†^	MAX^†^
Population	Proportion of population aged under 15 years	0.115	0.016	0.073	0.107	0.115	0.124	0.172
	Proportion of population aged 65 years and over	0.325	0.059	0.177	0.285	0.322	0.364	0.487
	Proportion of population change	–0.036	0.039	–0.113	–0.065	–0.037	–0.011	0.101
	Population density	1199	2837	12	88	240	676	18,939
	Proportion of population in densely inhabited districts	0.435	0.301	0.000	0.167	0.410	0.656	1.000
	Proportion of daytime to nighttime population	0.989	0.139	0.791	0.966	0.992	1.003	3.345
	Proportion of internal migrants from other municipalities	0.033	0.012	0.015	0.025	0.031	0.037	0.085
Household	Persons per household	2.314	0.199	1.660	2.169	2.321	2.451	2.826
	Proportion of single-person households (excluding households aged 65 years and over)	0.201	0.063	0.102	0.156	0.189	0.229	0.526
	Proportion of single-person households aged 65 years and over	0.138	0.035	0.062	0.111	0.130	0.161	0.240
	Proportion of nuclear households	0.552	0.042	0.347	0.530	0.553	0.576	0.705
	Proportion of double-income households	0.270	0.048	0.130	0.234	0.271	0.301	0.400
	Proportion of households living in a detached house	0.685	0.150	0.126	0.622	0.714	0.797	0.912
	Proportion of households living in government-owned rental housing	0.046	0.029	0.007	0.027	0.038	0.057	0.220
Industry & Work	Proportion of workers in primary industry	0.035	0.030	0.000	0.012	0.026	0.051	0.155
	Proportion of workers in secondary industry	0.117	0.037	0.042	0.091	0.111	0.140	0.219
	Proportion of workers in tertiary industry	0.311	0.026	0.252	0.293	0.309	0.329	0.416
	Proportion of working population	0.492	0.031	0.378	0.471	0.494	0.515	0.575
	Proportion of commuters using rail	0.055	0.069	0.000	0.013	0.026	0.058	0.296
	Proportion of commuters using their own vehicle	0.303	0.093	0.016	0.285	0.333	0.362	0.430
	Annual retail merchandise sales per capita	1,006,947	276,048	530,218	895,739	983,887	1,079,131	5,126,176
	Municipal residence tax per capita	52,019	17,379	30,886	41,060	48,343	56,713	192,724
Land utilization	Proportion of densely inhabited district areas	0.113	0.226	0.000	0.005	0.021	0.083	1.000
	Proportion of land area used for building	0.147	0.181	0.006	0.033	0.076	0.174	0.859
	Proportion of forest area	0.583	0.254	0.001	0.465	0.656	0.779	0.939
	Proportion of land area used for rice paddies and agriculture	0.172	0.114	0.000	0.090	0.149	0.232	0.607
	Proportion of depopulated area	0.487	0.384	0.000	0.000	0.517	0.859	1.062
	Number of pre-merger municipalities	10.033	5.308	1.000	6.000	9.000	13.000	28.000

^†^The abbreviations (Mean, SD, MIN, Q1, Q2, Q3, MAX) are as follows

*Mean* Mean Value, *SD* Standard Deviation, *MIN* Minimum Value, *Q1* First Quartile, *Q2* Second Quartile, *Q3* Third Quartile, *MAX* Maximum Value

**Table 4:** Reasons for the selection of the 28 variables related to regional characteristics

Category	Variable	Reason for the selection
Population	Proportion of population aged under 15 years	The demand for medical care is expected to increase with the number of children
	Proportion of population aged 65 years and over	The demand for medical and care increases with the number of older adults
	Proportion of population change	The supply of medical and care resources changes with the size of the population
	Population density	The higher the population density, the larger the population served by a single medical and care supply base and the more efficient the medical and care supply can be
	Proportion of population in densely inhabited districts	
	Proportion of daytime to nighttime population	The supply of medical and care resources is expected to change with changes in daytime and nighttime populations
	Proportion of internal migrants from other municipalities	The supply of medical and care resources changes with the size of the population
Household	Persons per household	A higher number of persons per household is expected to result in a higher percentage of persons living together and a lower demand for inpatient and institutional care
	Proportion of single-person households (excluding households aged 65 years and over)	The demand for hospitalization is expected to be higher for single-person households
	Proportion of single-person households aged 65 years and over	The demand for hospitalization and institutional care increases with a greater number of older single-person households
	Proportion of nuclear households	A higher percentage of nuclear households is expected to result in a higher percentage of persons living together and a lower demand for inpatient and institutional care
	Proportion of double-income households	A higher percentage of double-income households is expected to result in a higher percentage of persons living together at home and a lower demand for inpatient and institutional care
	Proportion of households living in a detached house	A higher percentage of households living in detached houses is expected to result in a less urbanized region, and thus in a situation where a single medical and care supply base cannot serve a large population or provide efficient medical and care supply
	Proportion of households living in government-owned rental housing	A higher percentage of households living in government-owned rental housing is expected to result in a higher percentage of low-wage earners and a higher demand for inpatient and institutional care
Industry & Work	Proportion of workers in primary industry	The supply of medical and care resources is expected to differ with the industrial composition
	Proportion of workers in secondary industry	
	Proportion of workers in tertiary industry	
	Proportion of working population	A higher percentage of the working population results in lower percentages of children and older populations and a lower demand for inpatient and institutional care
	Proportion of commuters using rail	A higher percentage of commuters using railways results in a more urbanized region, where a single medical and care supply base can serve a large population and medical and care supply is efficient
	Proportion of commuters using their own vehicle	A higher percentage of commuters using their own vehicle results in a less urbanized region, where a single medical and care supply base cannot serve a large population and medical and care supply is inefficient
	Annual retail merchandise sales per capita	Active purchasing behavior suggests more disposable income, which is related to health and thus affects the medical and care supply system
	Municipal residence tax per capita	A higher municipal residence tax suggests more income, which is related to health and thus affects the medical and care supply system
Land utilization	Proportion of densely inhabited district areas	A higher population density suggests a situation where a single medical and care supply base can serve a large population and medical and care supply is efficient
	Proportion of land area used for building	A higher percentage of land area used for building suggests a more urbanized region where a single medical and care supply base can serve a large population and medical and care supply is efficient
	Proportion of forest area	A higher percentage of forest area suggests a less urbanized region where a single medical and care supply base cannot serve a large population and medical and care supply is inefficient
	Proportion of land area used for rice paddies and agriculture	A higher percentage of land area used for rice paddies and agriculture suggests a less urbanized region where a single medical and care supply base cannot serve a large population and medical and care supply is inefficient
	Proportion of depopulated area	A higher percentage of depopulated area suggests a situation where a single medical and care supply base cannot serve a large population and medical and care supply is inefficient
	Number of pre-merger municipalities	A larger number of previous municipalities suggests dispersed medical and care supply bases before the merger and inefficient medical and care supply

The method of grouping SMAs by PCA was as follows. Initially, a PCA was performed to summarize the variables in Table 2. The number of PCs was determined from a parallel analysis, and a promax rotation was used to facilitate interpretation of the solution. Finally, the PC scores for all 334 SMAs were sorted in descending order and the SMAs were divided into top- and bottom-half groups. In the present study, a preferable method of analysis may have been to perform a detailed grouping based on a cluster analysis utilizing PC scores, as done in the study by Miyake et al. [26]. However, due to the smaller sample size in this method, multigroup analysis and SEM with such a detailed grouping often yields errors such as negative estimates of error variance. Therefore, grouping into two was assumed to be the maximum limit. Thus, the sample size for each group was 167. These PCA and parallel analyses were conducted using the “fa.parallel” and “principal” functions from version 2.2.9 of the “psych” package in R version 4.2.1 [32, 33]. The maximum likelihood method was used to estimate the PC loadings.

### Second phase: SEM for building the model of the associated structure of the distribution of medical and care resources in all of Japan

In total, there were 15 variables of the distribution of medical and care resources used in the second phase. These variables can be aggregated by SMA, with reference to the study by Kusunoki et al. [23], and are presented in Table 5 [34–36]. The summary statistics are presented in Table 6. The variables are classified into the following five categories: hospital re-sources, clinic resources, care resources, home medical care and home care resources, and other medical resources. Among these valuables, in Table 5, “Hospital beds for long-term hospitalization (per 10,000 people),” “Capacities of nursing homes (per 10,000 people),” “Workers in nursing homes (per 10,000 people),” and “Workers in home medical care and home care (per 10,000 people)” are aggregated resources that have similar characteristics regarding medical and care provision. An unaggregated data analysis by SEM failed to yield goodness-of-fit indexes within the acceptable range because of the large number of observed variables and multicollinearity. Therefore, the aggregation of the aforementioned variables is the maximum limit. Population data, which were obtained from the “Population Census” data for 2020 [25], were used to calculate the variables presented in Table 5.

**Table 5:** The 15 variables related to medical and care resources

Category	Variable	Calculation method	Source
Hospital resources	General hospital beds (per 10,000 people)	Number of “general hospital beds” / (population/10,000)	[34]
	Hospital beds for long-term care (per 10,000 people)	Number of “long-term hospital care beds” and “hospital psychiatric beds” / (population/10,000)	
	Hospital physicians (per 10,000 people)	Number of “hospital physicians” / (population/10,000)	
	Hospital nurses (per 10,000 people)	Number of “hospital nurses” / (population/10,000)	
	Hospital assistant nurses (per 10,000 people)	Number of “hospital assistant nurses” / (population/10,000)	
Clinic resources	Clinics without beds (per 10,000 people)	Number of “clinics without beds” / (population/10,000)	[34]
	Clinic beds (per 10,000 people)	Number of “clinic beds” (excluding clinic long-term care beds) / (population/10,000)	
	Clinic long-term care beds (per 10,000 people)	Number of “clinic long-term care beds” / (population/10,000)	
	Clinic physicians (per 10,000 people)	Number of “clinic physicians” / (population/10,000)	
	Clinic nurses (per 10,000 people)	Number of “clinic nurses” / (population/10,000)	
	Clinic assistant nurses (per 10,000 people)	Number of “clinic assistant nurses” / (population/10,000)	
Care resources	Capacities of nursing homes (per 10,000 people)	Capacities of “nursing homes” / (population/10,000)	[35]
	Workers in nursing homes (per 10,000 people)	Number of “nurses” and “staff members” in “nursing homes” / (population/10,000)	
Home medical care and home care resources	Workers in home medical care and home care(per 10,000 people)	Number of “nurses” and “staff members” in “home medical care” and “home care” / (population/10,000)	[35]
Other medical resources	Universities with a faculty of medicine	Number of “universities with a faculty of medicine”	[36]

**Table 6:** Summary statistics of the 15 variables related to medical and care resources

Category	Variable	mean^†^	SD^†^	MIN^†^	Q1^†^	Q2^†^	Q3^†^	MAX^†^
Hospital resources	General hospital beds (per 10,000 people)	76.366	22.940	21.258	59.544	73.098	90.612	169.916
	Hospital beds for long-term care (per 10,000 people)	61.346	35.933	0.000	35.484	52.552	76.763	281.538
	Hospital physicians (per 10,000 people)	13.253	5.973	2.453	9.452	12.100	15.119	62.815
	Hospital nurses (per 10,000 people)	68.009	20.248	12.101	52.812	66.253	82.672	140.001
	Hospital assistant nurses (per 10,000 people)	10.239	6.985	0.684	5.239	8.225	13.403	42.500
Clinic resources	Clinics without beds (per 10,000 people)	7.310	1.922	2.787	6.120	7.009	8.261	26.259
	Clinic beds (per 10,000 people)	9.389	8.719	0.000	3.465	6.369	11.779	50.354
	Clinic long-term care beds (per 10,000 people)	0.918	1.458	0.000	0.000	0.276	1.339	12.876
	Clinic physicians (per 10,000 people)	7.473	2.328	1.497	6.040	7.394	8.583	29.561
	Clinic nurses (per 10,000 people)	12.373	4.116	4.319	9.779	11.923	14.412	47.580
	Clinic assistant nurses (per 10,000 people)	9.187	5.268	1.747	5.253	8.071	11.916	30.923
Care resources	Capacities of nursing homes (per 10,000 people)	155.052	44.211	61.412	124.073	150.392	179.421	319.266
	Workers in nursing homes (per 10,000 people)	145.586	47.555	48.225	110.481	143.452	177.789	308.726
Home medical care and home care resources	Workers in home medical care and home care (per 10,000 people)	22.489	10.049	7.742	15.789	20.425	26.340	79.268

^†^The abbreviations (Mean, SD, MIN, Q1, Q2, Q3, MAX) are as follows

*Mean* Mean Value, *SD* Standard Deviation, *MIN* Minimum Value, *Q1* First Quartile, *Q2* Second Quartile, *Q3* Third Quartile, *MAX* Maximum Value

The method for building the model of the associated structure of the distribution of medical and care resources in the 334 SMAs nationwide by SEM was as follows. Initially, to formulate a hypothesis about this associated structure, an exploratory factor analysis (EFA) using the variables in Table 5 was performed. The number of factors was determined from a parallel analysis, and promax rotation was used. Subsequently, based on the results, we identified latent factors behind the variables in Table 5 from the obtained factors and formulated hypotheses regarding the relationships among them. Finally, SEM was used to test this hypothesis and to model the associated structure. The factor analysis and parallel analyses were conducted using the “fa.parallel” and “fa” functions from version 2.2.9 of the “psych” package in R version 4.2.1 [32, 33]. The maximum likelihood method was used to estimate the factor loadings. The SEM and multigroup analysis were conducted using the “sem” function from version 0.6.12 of the “lavaan” package in R version 4.2.1 [33, 37]. To estimate the population for these analyses, a diagonal weighted least squares method was used. This method employs a highly robust diagonal matrix to ensure accurate estimation results for non-multivariate normal data, rendering them suitable for utilizing the variables in Table 5 for medical and care resources. While the SEM and multigroup analysis were also conducted using the maximum likelihood method, which used PC and factor analyses, the values of the goodness-of-fit indexes utilized in this study were outside the tolerance limits. For the SEM and multigroup analysis described below, the goodness-of-fit indexes adopted in this study were the root mean square error of approximation (RMSEA), the comparative fit index (CFI), and the Tucker–Lewis index (TFI), the standardized root mean square residual (SRMR), and the *p*-value(χ^2^), which can be given by the “sem “ function. The RMSEA shows a high goodness-of-fit when the value is less than 0.05, and a low fit when the value is above 0.10. The CFI and TLI show a high goodness-of-fit when the values are more than 0.95. The *p*-value (χ^2^) indicates the probability of the test of the null hypothesis by the χ^2^ value that the model constructed fits the sample in SEM; thus, a higher probability indicates a higher goodness-of-fit. However, because the null hypothesis is more likely to be rejected as the sample size increases in SEM, the value of χ^2^ divided by the degrees of freedom (df), introduced by Schermelleh-Engel et al. [38], was used in this study. This value, “χ^2^/df “ shows a high goodness-of-fit when the value is less than 2.

### Third phase: multigroup analysis for comparing the model of each group

A multigroup analysis was conducted using the obtained structural model with the SMAs grouped by regional characteristics as the sample. However, when a sample size is halved, as in this case, a solution may not be obtained owing to the deterioration of the model’s goodness-of-fit index in the multigroup analysis. In such cases, a multigroup analysis is conducted after adding constraints to fix the error variance of specific variables to the obtained structural model. It is also expected that, even with these constraints, a solution to the multigroup analysis may not be obtained. In these cases, SEM is performed for each group, with the same arrangement of variables, but with constraints to fix the error variance of particular variables.

## Results

### PCA

**Fig. 1:**
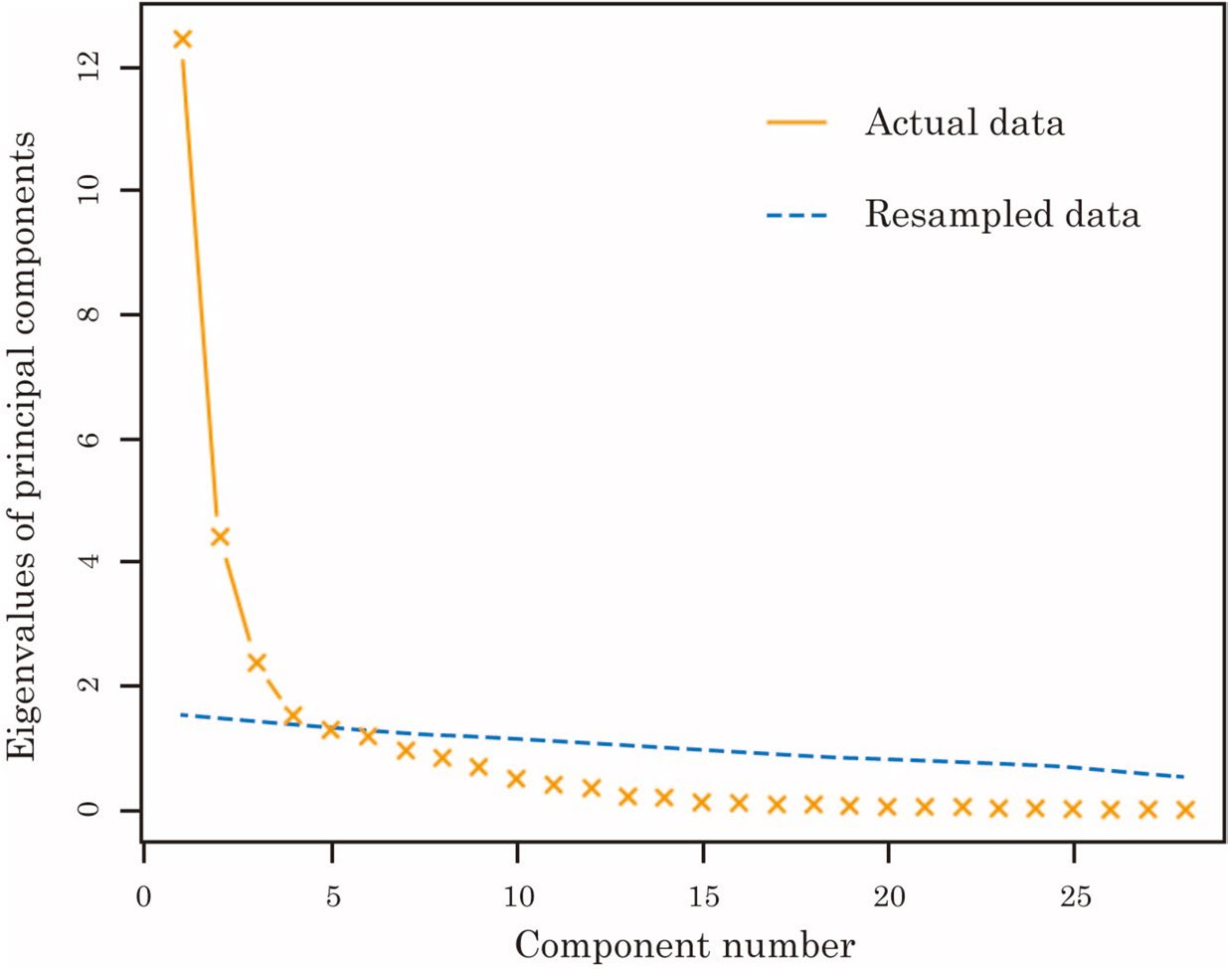
Results of the parallel analysis for the principal component analysis

Initially, the parallel analysis suggested that four PCs were suitable, as shown in Fig. 1. Subsequently, we interpreted the outcomes of the PCA in the following order of the contribution ratio, as shown in Table 7.

1. PC 1, which we interpreted as “urbanicity,” features high PC loadings for “population density” and other variables related to population concentration.
2. PC 2, which we interpreted as “middle-density regionality,” features high PC loadings for “Proportion of households living in a detached house” and “Proportion of commuters using their own vehicle.” The definition of a middle-density region used in this study was an area with a medium population density where many of the houses are detached and the primary means of transportation is the vehicle. Therefore, large cities with an extremely high population density where the primary means of transportation is railways and depopulated areas with an extremely low population density were not included in the middle-density region.
3. PC 3, which we interpreted as “workplace regionality,” features high PC loadings for “Proportion of daytime to nighttime population” and “annual retail merchandise sales per capita.”
4. PC 4, which we interpreted as “childcare regionality,” features high PC loadings for “Proportion of population aged under 15 years” and “Proportion of nuclear households.”

**Table 7:** Results of the principal component analysis

Category	Variable	Principal component loading			
		Urbanicity	Middle-density regionality	Workplace regionality	Childcare regionality
Population	Proportion of population aged under 15 years	0.47	–0.11	–0.10	0.73
	Proportion of population aged 65 years and over	–0.93	–0.05	–0.07	–0.32
	Proportion of population change	0.93	0.00	0.04	0.29
	Population density	0.73	0.01	0.16	–0.51
	Proportion of daytime to nighttime population	–0.03	0.02	0.86	–0.02
	Proportion of population in densely inhabited districts	0.78	–0.26	–0.09	0.08
	Proportion of internal migrants from other municipalities	0.53	–0.47	0.22	0.12
Household	Persons per household	0.09	0.89	–0.13	0.05
	Proportion of single-person households (excluding households aged 65 years and over)	0.59	–0.35	0.37	0.08
	Proportion of single-person households aged 65 years and over	–0.87	–0.56	–0.10	–0.25
	Proportion of nuclear households	0.15	–0.02	–0.68	0.37
	Proportion of double-income households	–0.20	0.85	0.07	–0.01
	Proportion of households living in a detached house	–0.70	0.42	–0.13	–0.08
	Proportion of households living in a government-owned rental housing	–0.38	–0.71	–0.10	–0.05
Industry & Work	Proportion of workers in primary industry	–0.76	–0.04	0.16	–0.09
	Proportion of workers in secondary industry	0.18	0.95	0.02	–0.07
	Proportion of workers in tertiary industry	0.15	–0.47	0.16	0.08
	Proportion of working population	–0.30	0.69	0.28	–0.07
	Proportion of commuters using rail	0.87	–0.01	–0.17	–0.36
	Proportion of commuters using their own vehicle	–0.60	0.43	0.13	0.30
	Annual retail merchandise sales per capita	0.15	0.03	0.86	0.04
	Municipal residence tax per capita	0.79	0.07	0.37	–0.19
Land utilization	Proportion of densely inhabited district areas	0.80	–0.02	0.03	–0.46
	Proportion of land area used for building	0.91	0.05	–0.08	–0.34
	Proportion of forest area	–0.85	–0.06	0.10	0.01
	Proportion of land area used for rice paddies and agriculture	0.13	0.16	–0.10	0.50
	Proportion of depopulated area	–0.92	–0.16	0.11	–0.14
	Number of pre-merger municipalities	–0.18	–0.07	0.22	0.42
Information volume	Urbanicity	Middle-density regionality	Workplace regionality	Childcare regionality	
Sum of squared loadings	10.9	4.90	2.69	2.31	
Contribution rate	0.39	0.17	0.10	0.08	
Cumulative contribution rate	0.39	0.56	0.66	0.74	
Correlation coefficient between principal components	Urbanicity	Middle-density regionality	Workplace regionality	Childcare regionality	
Urbanicity	-	–0.37	0.18	–0.05	
Middle-density regionality		-	–0.21	0.42	
Workplace regionality			-	–0.08	

Because promax rotation was used in the PCA, correlations were found between the obtained components. A slight negative correlation was seen between “urbanicity” and “middle-density regionality.” This could be because of the substantially higher population concentrations in urban areas and the middle concentrations in middle-density regions. A slight positive correlation was found between “middle-density regionality” and “childcare regionality.” This could be because middle-density regions are more suitable than urban areas for rearing children and have a higher proportion of households with children. A slight negative correlation was found between “workplace regionality” and “middle-density regionality,” and a slight positive correlation between “urbanicity” and “workplace regionality.” This could be due to commercial areas and business districts being biased toward locations with large population concentrations.

### Division of SMAs

**Fig. 2:**
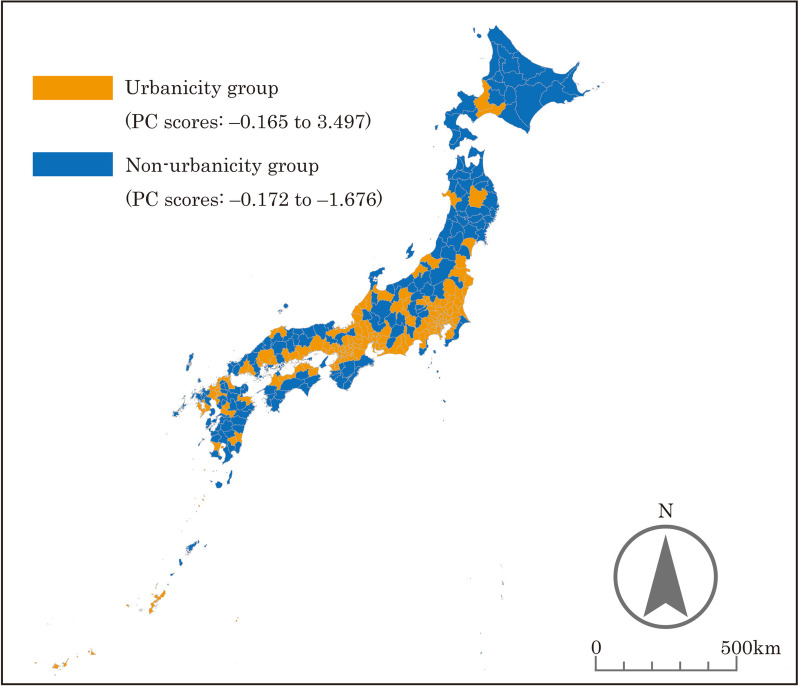
Results of the grouping by “urbanicity”

**Fig. 3:**
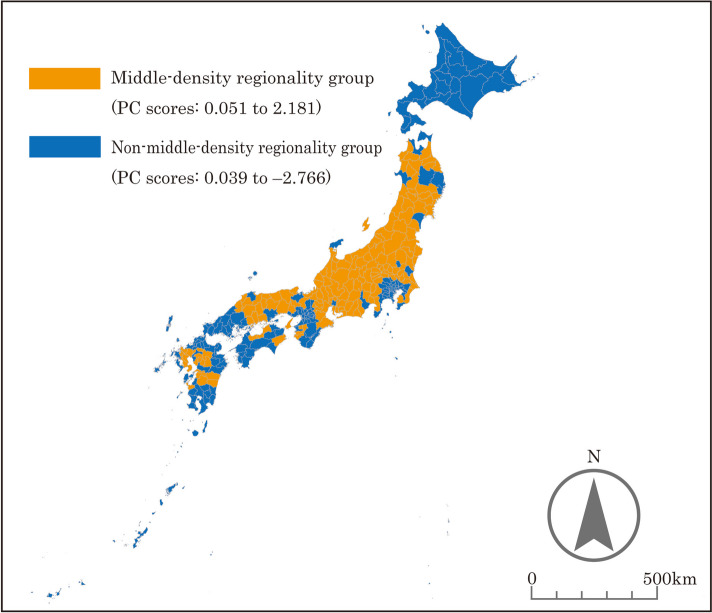
Results of the grouping by “middle-density regionality”

**Fig. 4:**
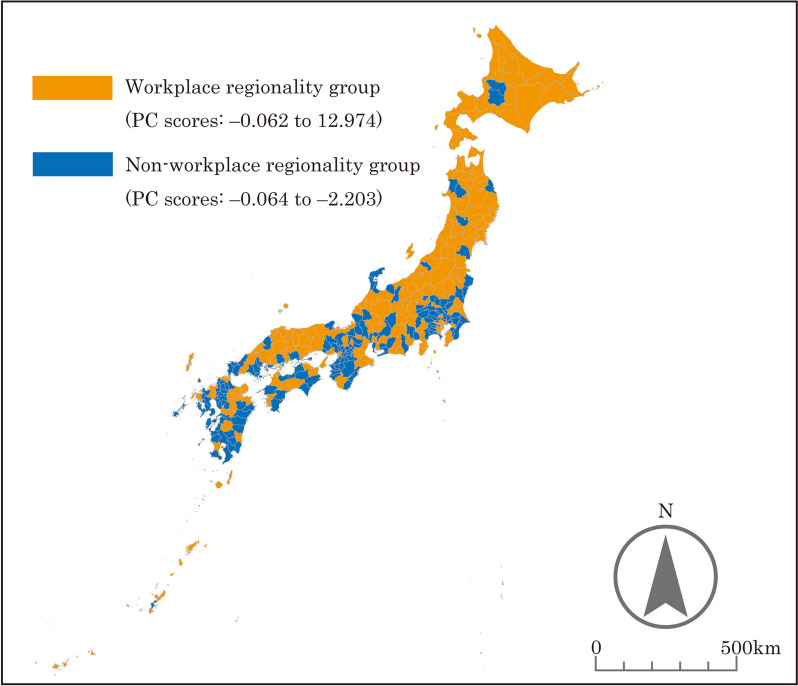
Results of the grouping by “workplace regionality”

**Fig. 5:**
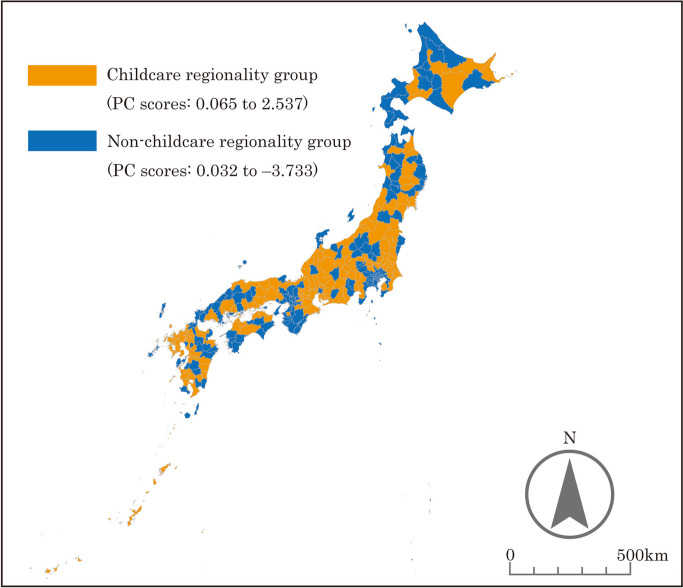
Results of the grouping by “childcare regionality”

Based on the PC scores, the SMAs nationwide were categorized into different groups. The results are presented in Figs. 2, 3, 4, 5, and the characteristics of each grouping are summarized below.

1. The grouping by “urbanicity” indicated that the areas along the Pacific Belt and Japan’s major cities (Tokyo, Osaka, Nagoya, Fukuoka, Sendai, and Sapporo) were classified as high “urbanicity” SMAs. In addition, SMAs with prefectural capitals tended to be classified as high “urbanicity” areas. The reason why some SMAs, such as Okinawa Prefecture and its surrounding islands far from Honshu mainland, were classified as high “urbanicity” is seemingly due to their high population density.
2. As defined earlier, the grouping by “middle-density regionality” indicated that the areas that are not included in Japan’s major cities with an extremely high population density (Tokyo, Osaka, Nagoya, Fukuoka, Sendai, and Sapporo) or depopulated areas with an extremely low population density were classified as high “middle-density regionality” SMAs. SMAs classified as high “middle-density regionality” areas included urban and rural areas. This seems to be due to the presence of areas with a medium population density among urban areas and areas with a certain population size among rural areas. For this reason, SMAs consisting of Hokkaido Prefecture, where the population is concentrated in Sapporo, and several other large cities were classified as low “middle-density regionality” areas, even though most of the rest of the municipalities have a very low population density.
3. The grouping by “workplace regionality” indicated that the workplace regionality of Japan’s major cities was high, whereas that of the surrounding areas was low, as these areas primarily serve as “bedroom communities.”
4. The grouping by “childcare regionality” indicated that Japan’s major cities (other than Tokyo, Osaka, and Nagoya) were classified as areas with high childcare regionality, whereas remote areas were classified as areas with low childcare regionality.

### EFA

**Fig. 6:**
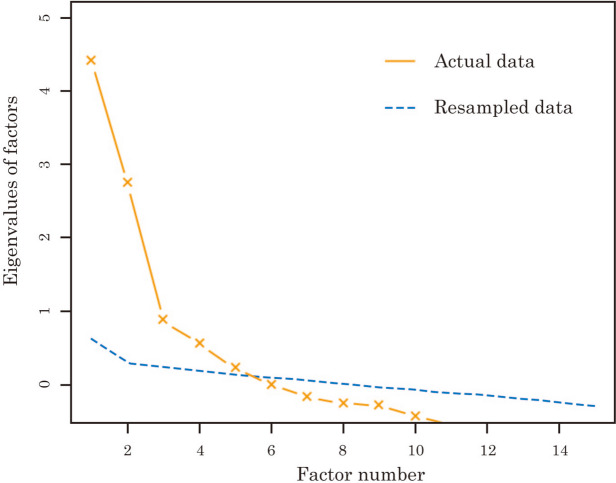
Results of the parallel analysis for the factor analysis

Initially, the results of the parallel analysis suggested that five factors were suitable, as shown in Fig. 6. Subsequently, we interpreted the outcomes of the EFA in the following order of the contribution ratio, as shown in Table 8.

1. Factor 1, namely “Clinic care abundance (without beds),” was determined to have a high factor loading for “Clinics without beds (per 10,000 people),” indicating an abundance of clinics without beds.
2. Factor 2, namely “Clinic care abundance (with beds),” was determined to have high factor loadings for “Clinic beds (per 10,000 people)” and “Clinic long-term care beds (per 10,000 people),” indicating an abundance of clinics with beds.
3. Factor 3, namely “Nursing care abundance,” was determined to have high factor loadings for “Capacities of nursing home (per 10,000 people)” and “Workers in nursing homes (per 10,000 people),” indicating an abundance of nursing homes.
4. Factor 4, namely “Acute care abundance,” was determined to have a high factor loading for “General hospital beds (per 10,000 people),” indicating an abundance of hospitals offering acute care.
5. Factor 5, namely “Long-term hospitalization care abundance,” was determined to have a high factor loading for “Hospital beds for long-term hospitalization,” indicating an abundance of hospitals providing long-term hospitalization care.

**Table 8:** Results of the factor analysis

Category	Variable	Factor loading				
		Clinic care abundance (without beds)	Clinic care abundance (with beds)	Nursing care abundance	Acute care abundance	Long-term hospitalization care abundance
Hospital resources	Hospital general beds (per 10,000 people)	–0.20	0.02	0.24	0.81	0.06
	Hospital beds for long-term hospitalization (per 10,000 people)	0.00	–0.01	–0.04	0.16	0.85
	Hospital physicians (per 10,000 people)	0.24	–0.08	–0.17	0.71	–0.06
	Hospital nurses (per 10,000 people)	–0.05	0.02	0.06	0.95	0.14
	Hospital assistant nurses (per 10,000 people)	0.01	0.01	–0.08	0.00	1.02
Clinic resources	Clinics without beds (per 10,000 people)	1.01	–0.32	0.31	–0.12	–0.02
	Clinic beds (per 10,000 people)	–0.14	1.03	–0.11	0.03	0.05
	Clinic long-term care beds (per 10,000 people)	–0.07	0.78	0.08	–0.01	–0.07
	Clinic physicians (per 10,000 people)	1.05	0.03	–0.25	–0.15	0.17
	Clinic nurses (per 10,000 people)	0.51	0.43	–0.05	0.08	–0.18
	Clinic assistant nurses (per 10,000 people)	0.16	0.52	0.10	–0.11	0.38
Care resources	Capacities of nursing home (per 10,000 people)	–0.01	–0.06	0.87	0.05	0.01
	Workers in nursing homes (per 10,000 people)	–0.05	0.05	1.03	0.10	–0.13
Home Medical care and home care resources	Workers in home medical care and home care (per 10,000 people)	0.26	0.06	0.27	0.08	0.03
Other medical resources	Universities with a faculty of medicine	0.35	–0.07	–0.33	0.29	–0.08
Information volume		Clinic care abundance (without beds)	Clinic care abundance (with beds)	Nursing care abundance	Acute care abundance	Long-term hospitalization care abundance
Sum of squared loadings		2.56	2.23	2.19	2.18	2.01
Contribution rate		0.17	0.15	0.15	0.15	0.13
Cumulative contribution rate		0.17	0.32	0.47	0.61	0.74
Correlation coefficient between factors		Clinic care abundance (without beds)	Clinic care abundance (with beds)	Nursing care abundance	Acute care abundance	Long-term hospitalization care abundance
Clinic care abundance (without beds)		-	0.25	–0.05	0.58	–0.08
Clinic care abundance (with beds)			-	0.39	0.32	0.54
Nursing care abundance				-	0.06	0.59
Acute care abundance					-	0.20

Table 8 shows the correlation coefficients of 0.5 or higher among some of the factors. The factor “Clinic care abundance (without beds)” was positively correlated with the factor “Acute care abundance,” both of which affect the provision of medical ser-vices related to short-term hospitalization and outpatient visits. “Nursing care abundance” was positively correlated with both “Clinic care abundance (with beds)” and “Long-term hospitalization care abundance,” which affect the provision of medical and care services related to long-term hospitalization and nursing home care.

### Hypotheses based on EFA results and validation by SEM

Based on the results discussed above, we formulated hypotheses regarding the associated structure of the distribution of medical and care resources in this study. Our first hypothesis concerns the latent factors that cause the distribution of medical or care resources. Local demand determines the distribution of both resources, but with different constraints on the supply of each. The distribution of hospital and clinic beds, classified as medical resources, is subject to strong restrictions because of the standard number of beds being determined based on the population by age group and bed utilization in a specific SMA to prevent excessive resource supply. Additionally, the MHLW induces prefectures to enhance home medical care, home care, and care in nursing homes when they formulate regional medical visions [39]. While nursing homes have personnel standards and other requirements, which are classified as care resources, they have fewer restrictions on their expansion than on medical resources. Therefore, the degree of enhancement of care resources is influenced by not only the degree of enhancement of medical resources, but also regional characteristics such as land prices and the number of workers needed to operate nursing homes. By contrast, the degree of medical resources is more directly influenced by the degree of care resources, which affects bed utilization, than by these regional characteristics, as the standard number of hospital beds is constrained. Based on these inferences, and in light of the fact that the SEM in this study did not deal with regional characteristics, we constructed a model that assumes “Nursing care abundance” as the cause.

Our second hypothesis concerns the causal relationship among the latent factors related to medical resources. The results of the EFA indicated a correlation between the two latent factors related to short-term hospitalization as well as outpatient visits, and the two latent factors related to long-term hospitalization, respectively. Therefore, we investigated whether the causal latent factor is related to short-term hospitalization and outpatient visits, or to long-term hospitalization. To formulate this hypothesis, we referred to Innami [40], who discussed social hospitalization. Innami considered that there is a trend for older adults to be admitted to a hospital with long-term care beds as a substitute for fully occupied nursing homes, and to be accepted to a hospital with general beds as a substitute for fully occupied long-term care beds. From this perspective, we hypothesized that “Long-term hospitalization care abundance” and “Clinic care abundance (with beds)” related to long-term hospitalization affect “Acute care abundance” and “Clinic care abundance (without beds)” related to short-term hospitalization and outpatient visits.

**Fig. 7:**
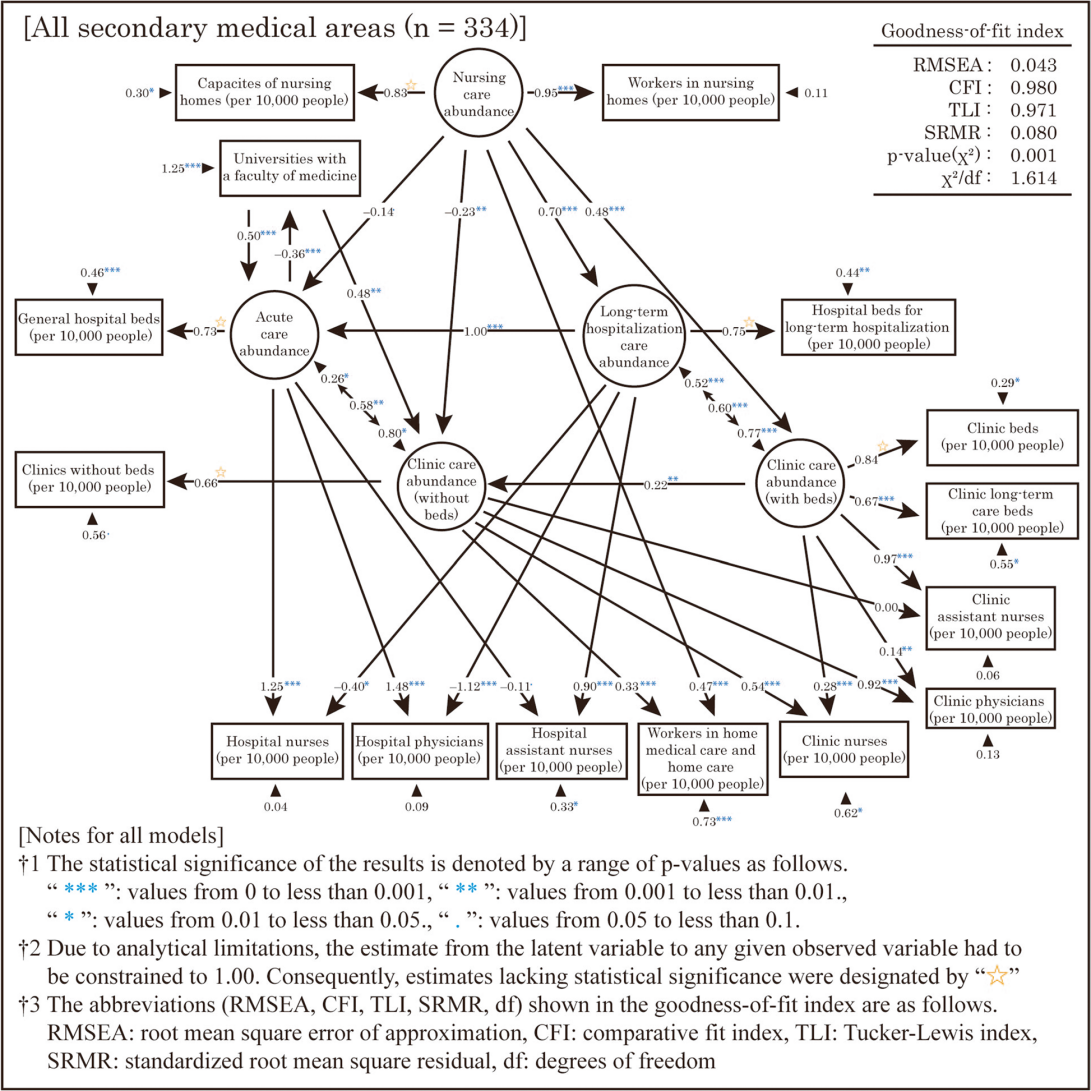
Resulting model of the structural equation modeling

The third hypothesis is related to the relationship between latent and observed variables, where each latent variable is defined based on the findings of the EFA. To facilitate the interpretation of the SEM solution, we assumed that only one of the latent variables was responsible for the observed variables, namely, the number of hospital beds, number of clinic beds, and nursing home capacity. However, in the case of the observed variables related to medical professionals, such as physicians, nurses, and assistant nurses, multiple latent variables were assumed to be involved, even when the factor loadings on the factors considered as latent variables were low. In addition, we assumed that “Nursing care abundance” and “Clinic care abundance (without beds)” were behind the observed variable “Workers in home medical care and home care (per 10,000 people)” based on the results of the EFA. However, the observed variable “Universities with a faculty of medicine” was not expected to be affected by the latent variables assumed in this study. Therefore, we assumed that this variable exerted an influence on “Clinic care abundance (without beds)” and “Acute care abundance” based on the findings of the EFA. Finally, Fig. 7 depicts the resulting SEM used to test the above hypotheses. Based on the goodness-of-fit indexes, the model showed an acceptable level of fit. Note that in addition to the hypothesis of this study, by employing SEM, the assumption that latent factors related to medical resources are the cause could be tested. Therefore, analyses were conducted assuming that both “Acute care abundance” and “Long-term hospitalization care abundance” were causes. However, these analyses generated cases that were not acceptable from the goodness-of-fit indexes used in this study and cases in which the solutions did not converge.

### Multigroup analysis

The results of the multigroup analysis for each of the four groupings shown in the "Division of SMAs" are presented below.

**Fig. 8:**
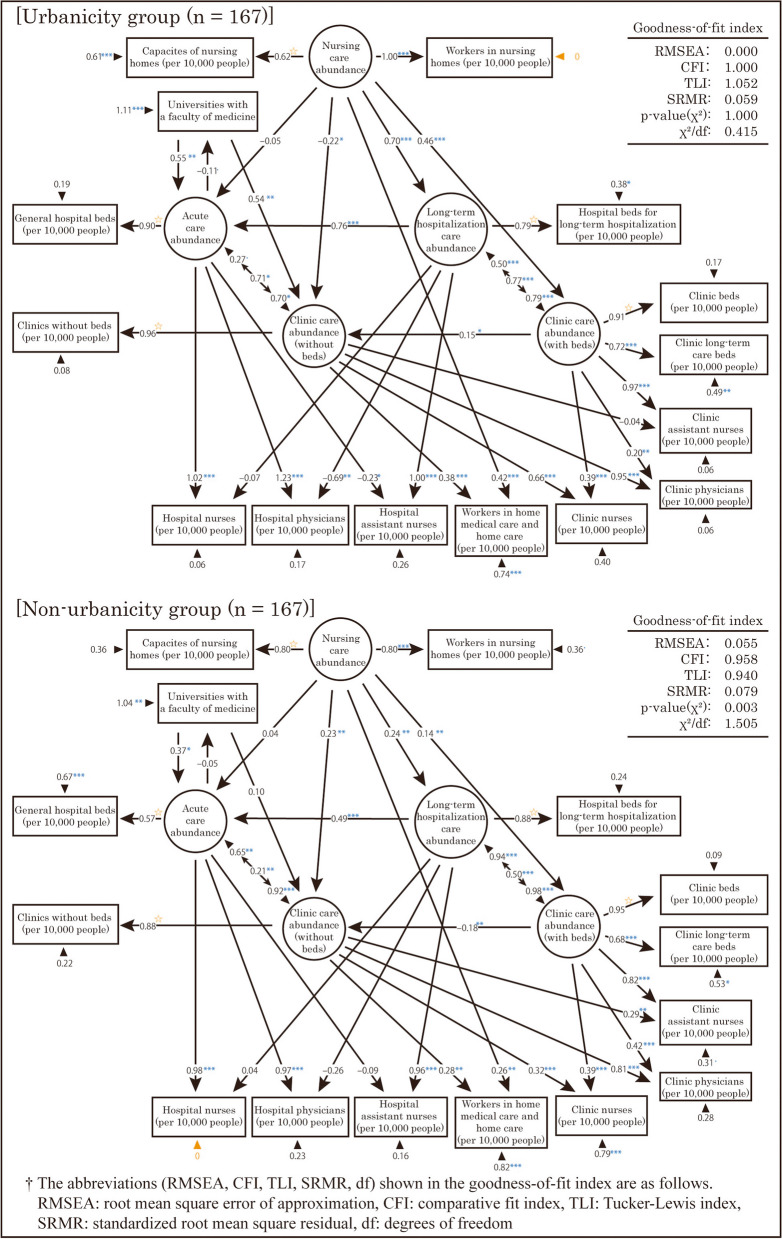
Resulting model of the structural equation modeling by “urbanicity” and “non-urbanicity”

First, a multigroup analysis was performed with two groups divided by “urbanicity.” However, in the analysis, the error variances of the observed variables “Hospital nurses (per 10,000 people)” and “Workers in nursing homes (per 10,000 people)” were negative. Therefore, we added a constraint to fix these error variances at 0. However, when this constraint was applied, no solution was obtained because the covariance matrix of the residuals of the observed variables was not positively definite. As a result, SEM was performed for each of the two groups with a common arrangement of variables, but with constraints to fix the error variances of the variables in question in each group; the results are shown in Fig. 8. Based on the goodness-of-fit indexes shown in this figure, the model showed an acceptable level of fit. Strictly speaking, the two models obtained had different structures and should be interpreted with this in mind.

The standardized partial regression coefficients for the same path differed by more than 0.3 between the two models at the following locations.

1. The path coefficients from “Nursing care abundance” to “Long-term hospitalization care abundance” and “Clinic care abundance (with beds)” were higher in the urbanicity group.
2. The path coefficient from “Nursing care abundance” to “Clinic care abundance (without beds)” was negative for the urbanicity group, but positive for the non-urbanicity group.
3. The path coefficient from “Clinic care abundance (with beds)” to “Clinic care abundance (without beds)” was positive for the urbanicity group, but negative for the non-urbanicity group.
4. The path coefficient for the covariate relationship between “Acute care abundance” and “Clinic care abundance (without beds)” was higher in the urbanicity group.
5. The path coefficient from “Universities with a faculty of medicine” to “Clinic care abundance (without beds)” was higher in the urbanicity group.
6. The path coefficient from “Long-term hospitalization care abundance” to “Hospital physicians (per 10,000 people)” was negative in both groups, with a higher absolute value in the urbanicity group.
7. The path coefficient from “Clinic care abundance (without beds)” to “Clinic nurses (per 10,000 people)” was higher in the urbanicity group. Conversely, the path co-efficient from “Clinic care abundance (without beds)” to “Clinic assistant nurses (per 10,000 people)” was negative for the urbanicity group and positive for the non-urbanicity group.

**Fig. 9:**
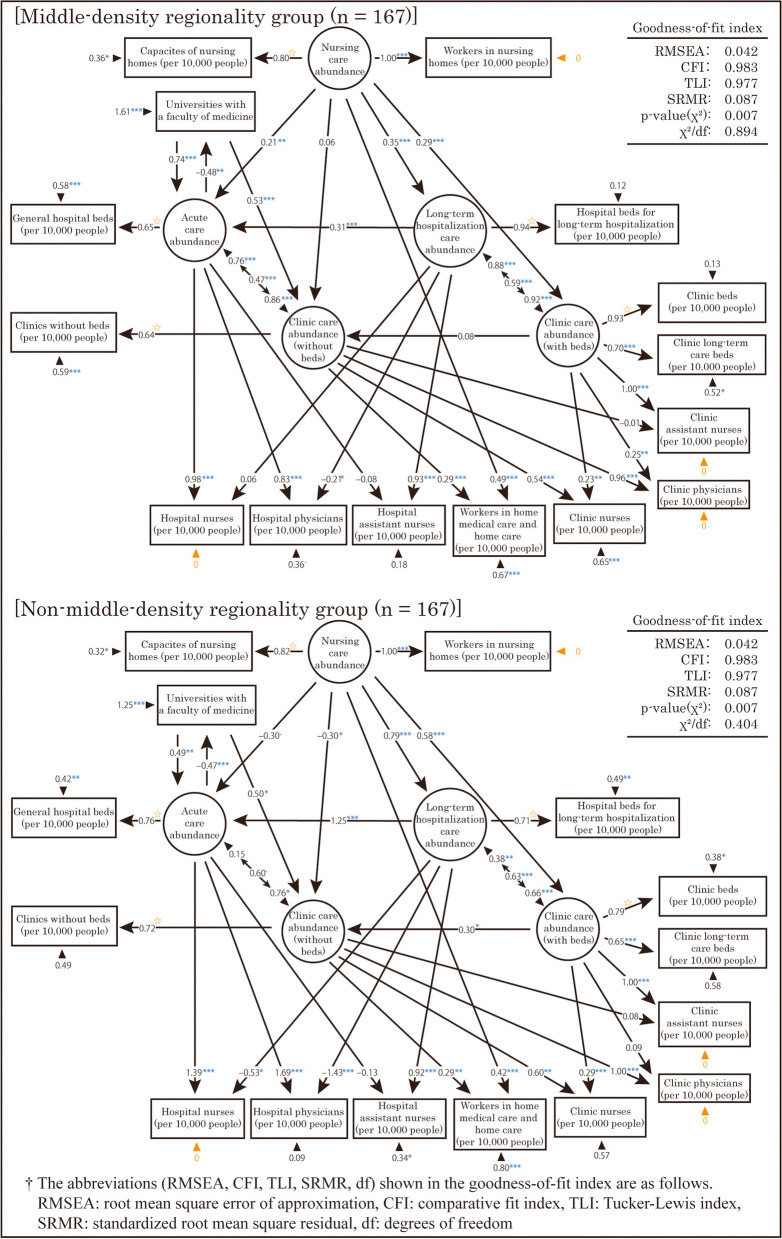
Resulting model of the structural equation modeling by “middle-density regionality” and “non-middle-density regionality”

Second, a multigroup analysis was performed with two groups categorized by “middle-density regionality.” However, in this analysis, the error variances of the observed variables “Hospital nurses (per 10,000 people),” “Workers in nursing homes (per 10,000 people),” “Clinic physicians (per 10,000 people),” and “Clinic assistant nurses (per 10,000 people)” were found to be negative. Thus, constraints were added to fix the error variances of these variables to 0. The results are displayed in Fig. 9. Based on the goodness-of-fit indexes shown in this figure, the model showed an acceptable level of fit.

The standardized partial regression coefficients for the same path differed by more than 0.3 between the two models at the following locations.

1. The path coefficients from “Nursing care abundance” to “Acute care abundance” and “Clinic care abundance (without beds)” were positive in the middle-density regionality group, but negative in the non-middle-density regionality group. By contrast, the path coefficient from “Nursing care abundance” to “Long-term hospitalization care abundance” was low in the middle-density regionality group.
2. The path coefficient from “Long-term hospitalization care abundance” to “Acute care abundance” was high in the non-middle-density regionality group.
3. The path coefficients from “Acute care abundance” to “Hospital physicians (per 10,000 people)” and “Hospital nurses (per 10,000 people)” were lower in the middle-density regionality group. Conversely, the path coefficients from “Long-term hospitalization care abundance” to these observed variables were lower in the non-middle-density regionality group.

**Fig. 10:**
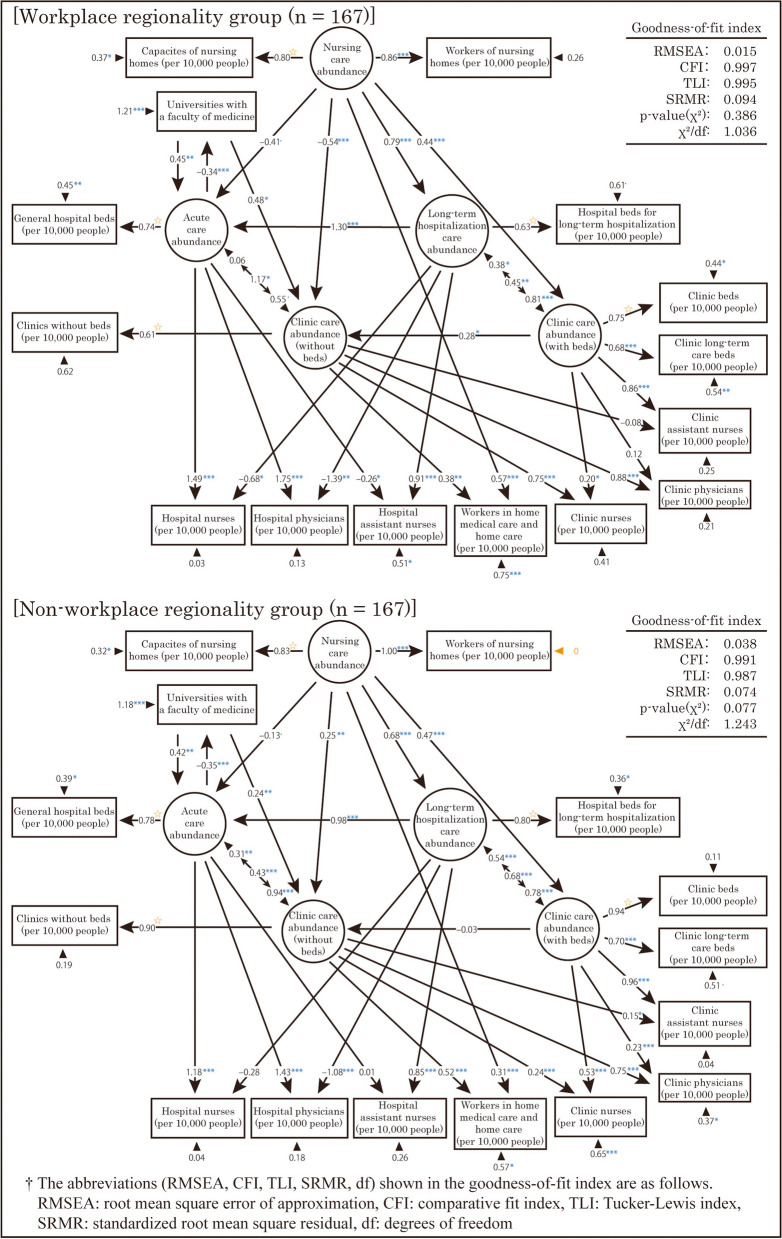
Resulting model of the structural equation modeling by “workplace regionality” and “non-workplace regionality”

Third, a multigroup analysis was performed with two groups divided by “workplace regionality.” However, in the analysis, the error variances of the observed variables “Workers in nursing homes (per 10,000 people)” was negative. Therefore, we added constraints to fix these error variances at 0. However, this resulted in no solution because of an optimizer error. As a result, SEM was performed for each of the two groups with a common arrangement of variables, but with constraints to fix the error variances of the variables in question in each group. The results are shown in Fig. 10. Based on the goodness-of-fit indexes shown in this figure, the model showed an acceptable level of fit. Strictly speaking, the two models obtained had different structures, and should be interpreted with this in mind.

The standardized partial regression coefficients for the same path differed by more than 0.3 between the two models at the following locations.

- (1) The path coefficient from “Nursing care abundance” to “Clinic care abundance (without beds)” was negative for the workplace regionality group, but positive for the non-workplace regionality group.
- (2) The path coefficients from “Acute care abundance” to “Hospital physicians (per 10,000 people)” and “Hospital nurses (per 10,000 people)” were higher in the workplace regionality group. In contrast, the path coefficients from “Long-term hospitalization care abundance” to these observed variables were lower in the workplace regionality group.
- (3) The path coefficients from “Clinic care abundance (without beds)” to “Clinic nurses (per 10,000 people)” were higher in the workplace regionality group. Conversely, the path coefficients from “Clinic care (without beds) abundance” to “Clinic assistant nurses (per 10,000 people)” were lower in the workplace regionality group.

**Fig. 11:**
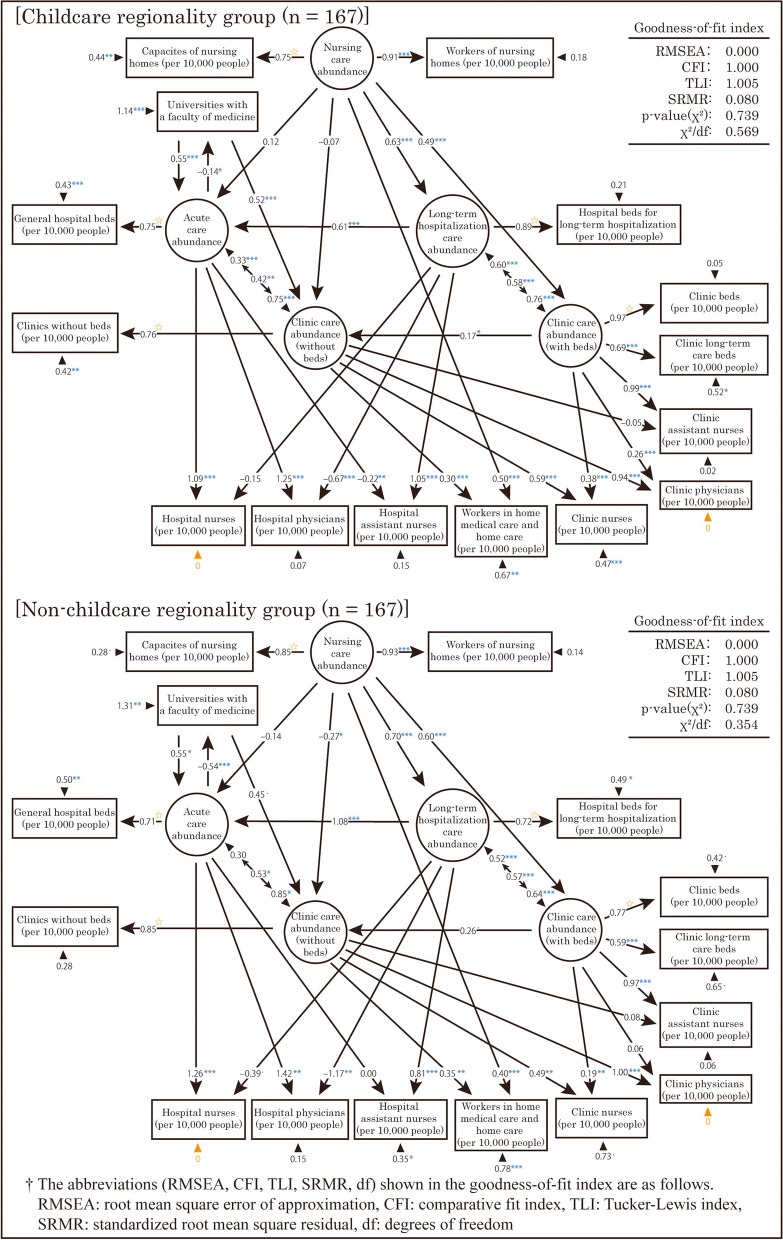
Resulting model of the structural equation modeling by “childcare regionality” and “non-childcare regionality”

Fourth, a multigroup analysis was performed with two groups based on “childcare regionality.” However, in this analysis, the error variances of the observed variables “Hospital nurses (per 10,000 people)” and “Clinic physicians (per 10,000 people)” were found to be negative. Thus, constraints were added to fix the error variances of these variables to 0. The results are displayed in Fig. 11. Based on the goodness-of-fit indexes shown in this figure, the model showed an acceptable level of fit.

The standardized partial regression coefficients for the same path differed by more than 0.3 between the two models at the following locations.

1. The path coefficient from “Acute care abundance” to “Universities with a faculty of medicine” was negative in both groups, but the absolute value was greater in the “non-childcare regionality” group.
2. The path coefficient from “Long-term hospitalization care abundance” to “Acute care abundance” was lower in the “childcare regionality” group.
3. The path coefficient from “Long-term hospitalization care” to “Hospital physicians (per 10,000 people)” was negative in both groups, and the absolute value was larger in the “non-childcare regionality” group.

## Discussion

### Distribution structure of medical and care resources in SMAs nationwide

The distribution structure of medical and care resources in SMAs nationwide is shown in Fig. 7. Our analysis suggests that “Nursing care abundance” has a slightly negative effect on “Acute care abundance” and “Clinic care abundance (without beds).” A previous study by Suzuki et al. [41] reported a weak negative correlation between medical and care expenditures. Although their study was limited to a few regions and used data from 2003 to 2007, our results confirm the validity of the model used. Our findings are also consistent with the MHLW’s policy to expand the use of nursing homes to reduce the number of general hospital beds. However, the results also indicate that the enhancement of nursing homes has only a slight effect on the reduction of general hospital beds and reduces the number of clinics without beds, which promotes home medical care services. Thus, our findings suggest that the MHLW’s goal of reducing hospital beds is difficult to achieve through nursing home enhancements alone.

Moreover, our results indicate that “Nursing care abundance” tends to increase “Long-term hospitalization care abundance” and “Clinic care abundance (with beds),” which implies that nursing homes alone are inadequate to meet the high demand for medical and care services, and therefore, must be supplemented with long-term hospitalization beds in hospitals and clinics. Additionally, “Long-term hospitalization care abundance” tends to increase “Acute care abundance,” and “Clinic care abundance (with beds)” tends to increase “Clinic care abundance (without beds).” Hence, our results support the consideration of Innami [40], previously mentioned in the section “Hypotheses based on EFA results and validation by SEM.”

“Long-term hospitalization care abundance” and “Clinic care abundance (with beds)” significantly increase only the number of assistant nurses. This suggests that associate nurses play a larger role in long-term hospitalization and clinic beds.

Moreover, “Universities with a faculty of medicine” tend to increase “Acute care abundance” and “Clinic care abundance (without beds).” A study by Kusunoki et al. [23] reported that the number of universities with a faculty of medicine increases the adequacy of acute care medical resources. Although their study used data on the distribution of medical resources in 2015, our results support the validity of the model used. Furthermore, our findings suggest that the location of universities with a faculty of medicine leads to increased fulfillment related to short-term hospitalization and outpatient visits in the surrounding areas.

### Distribution structure of medical and care resources considering regional characteristics

As previously stated, the subdivision of SMAs was based on PC scores for regional characteristics such as “urbanicity,” “middle-density regionality,” “workplace regionality,” and “childcare regionality,” which were determined through a PCA. “Workplace regionality” showed a slight correlation with “urbanicity,” while “childcare regionality” showed a slight correlation with “middle-density regionality.” Consequently, the differences in path coefficients for the distribution structure of medical and care resources across groups divided by the PCs with slight correlations were similar. Moreover, “workplace regionality” and “childcare regionality” showed low contribution rates. Therefore, the following discussion focuses on differences in the distribution structure of medical and care resources among each group divided by “urbanicity” and “middle-density regionality.”

### Analysis based on Urbanicity

Figure 8 shows insights into differences in the distribution structure of medical and care resources between the urbanicity and non-urbanicity groups. In the urbanicity group, “Nursing care abundance” tended to increase “Long-term hospitalization care abundance” and “Clinic care abundance (with beds).” This observation suggests that in urban areas, the demand for nursing homes is met by the supply of long-term hospitalization and clinic beds. The reason for this could be the unavailability of land required for expanding nursing homes in urban areas due to the limited land size and high land price.

Furthermore, in the urbanicity group, “Nursing care abundance” tended to reduce “Clinic care abundance (without beds),” whereas in the non-urbanicity group, it tended to increase it. This finding suggests that in the urbanicity group, the supply of nursing homes and clinics without beds is in a trade-off relationship with the demand for medical and care services among older adults. Conversely, it suggests that in the non-urbanicity group, the demand for medical and care services for older adults is met by clinics without beds instead of nursing homes. In Japan, primary care is often provided by the nearest medical institution from the patient’s home. The non-urbanicity group does not have an incentive to locate a large medical institution such as a hospital because of its small population size, so clinics that are relatively easy to establish play the primary care role. As a result, in the non-urbanicity group, primary care by clinics without beds and home care can meet the demand for nursing care. In addition, the non-urbanicity group may make demands for medical and care flow out to neighboring SMAs because their vast distance from large cities creates an area unsupplied with medical and care resources. However, the present study could not test this possibility because we did not use data on patient transfers between SMAs. For this reason, it is desirable to include data such as the proportion of outflow and inflow of patients per SMA published by the MHLW.

“Universities with a faculty of medicine” in the urbanicity group tended to increase “Clinic care abundance (without beds).” This trend may be attributed to the tendency for universities with a faculty of medicine to be located in urban areas, as indicated by the study by Kusunoki et al. [23].

Additionally, in the urbanicity group, “Long-term hospitalization care abundance” tended to decrease “Hospital physicians (per 10,000 people),” whereas “Acute care abundance” tended to increase “Hospital physicians (per 10,000 people).” Thus, in the urbanicity group, hospital physicians tended to be more available in acute care sections. This could be because physicians are concentrated in acute care owing to the existence of more universities with a faculty of medicine in the urbanicity group, where the supply of advanced acute care is greater compared with the non-urbanicity group.

By contrast, in the non-urbanicity group, “Clinic care abundance (without beds)” was less likely to increase “Clinic nurses (per 10,000 people),” but showed a strong tendency to increase “Clinic assistant nurses (per 10,000 people).” The path coefficients suggest that in the non-urbanicity group, the tendency of “Clinic care abundance (without beds)” to increase “Clinic nurses (per 10,000 people)” and “Clinic assistant nurses (per 10,000 people)” was similar, which suggests that associate nurses play a larger role in clinics without beds in non-urbanicity groups. Associate nurses have more limited duties than do nurses because they provide nursing care under the direction of physicians and nurses. Therefore, the quality of nursing care may be lower in the non-urbanicity group of clinics without beds than in the urbanicity group. However, the present study could not test this possibility because the discussion of the quality of medicine used only data such as the number of medical workers per population in each SMA. For this reason, it is desirable to include data such as the number of medical accidents as indicators of the quality of medicine in future work.

### Analysis based on middle-density regionality

Figure 9 provides insights into the distribution structure of medical and care resources between the middle and non-middle-density regionality groups. In the middle-density regionality group, “Nursing care abundance” appeared to increase “Acute care abundance” slightly. This suggests that the MHLW’s plan to reduce the number of general hospital beds by enhancing nursing homes may not produce favorable results in middle-density regionality areas. Here, we focus on two reports concerning the distribution of occupied nursing homes. The first report, by Oguro et al. [42], focused on sub-urban rural areas in Niigata city within an SMA in the middle-density regionality group. That study reported a slight excess of nursing homes compared with demand. The second report, by Asano et al. [43], concerns Toyohashi city within an SMA in the middle-density regionality group. That study reported an increasing trend of nursing homes in urbanization control areas as defined by the Japanese City Planning Act, where development is strictly restricted. Although those studies were based on historical data and specific areas smaller than SMAs, the results suggested that similar distribution patterns of nursing homes exist in municipalities within SMAs with high middle-density regionality areas nationwide. From the urban planning perspective, middle-density regionality areas are of concern with regard to urban sprawl. Thus, the expansion of nursing homes in these areas should be closely monitored not only for the reduction of hospital beds and the efficient provision of care, but also from the perspective of preventing overdevelopment.

In the non-middle-density regionality group, “Nursing care abundance” tended to reduce “Acute care abundance” and “Clinic care abundance (without beds).” Furthermore, “Nursing care abundance” tended to increase “Long-term hospitalization care abundance.” Thus, in non-middle-density regionality areas, compared with middle-density regionality areas, the demand for care is met by the supply of long-term hospitalization and clinic beds instead of nursing homes. According to the results of the PCA, non-middle-density regionality areas are typical urban or remote areas. In typically urban areas, this result is consistent with the associated structure of medical and care resources in the urbanicity group. A study by Nakazono et al. [44, 45] in remote areas reported fewer users of nursing homes due to a decline in the older population. A related study conducted by Tokito et al. [46] estimated that the amount of care service demand in remote areas tends to decrease with future changes in the older population. Although those studies were based on specific areas smaller than SMAs and used historical data, their results suggest a nationwide decline in demand for nursing homes in remote areas, which can lead to a disproportionate care supply. Consequently, supplemental medical and care supply with long-term hospitalization and clinic beds may meet the uneven demand for care in different locations.

In the non-middle-density regionality group, we observed strong tendencies for “Long-term hospitalization care abundance” to increase “Acute care abundance” and for “Nursing care abundance” to increase “Long-term hospitalization care abundance.” This result may be attributable to the consideration of Innami [40], previously mentioned in the section “Hypotheses based on EFA results and validation by SEM.” In addition, in the non-middle-density regionality group, “Long-term hospitalization care abundance” tended to reduce “Hospital physicians (per 10,000 people)” and “Hospital nurses (per 10,000 people).” This suggests that the quality of medicine related to long-term hospitalization is lower in the non-middle-density regionality group. In a typical urban area among the non-middle-density regions, this result is consistent with the associated structure of medical and care resources in the urbanicity group.

### Limitations

This study has some limitations in addition to those previously mentioned. Primarily, because this study was conducted in a cross-sectional setting, causal relationships could not be identified. A time series analysis, for example, would be desirable to resolve this issue. Second, this study was limited to discussing regional differences in medical and care resources solely based on supply-side factors using data on medical and care resources. However, studies by Ibuka et al. [47] and Jin et al. [48] reported that regional differences in medical and care resources are also dependent on demand-side factors. Therefore, it is desirable to include data generated by demand-side factors, such as medical and care costs based on receipt data. Based on the perspective of the related structure of medical and care resources according to regional characteristics obtained in the present study, these findings are expected to provide insights into factors that contribute to unnecessary medical and care provision, such as the physician-induced demand hypothesis [49] discussed in health economics.

## Conclusions

This study elucidated the distribution structure of medical and care resources in SMAs nationwide. Furthermore, it elucidated regional differences in the distribution of medical and care resources. The main findings of this study are summarized as follows.

First, with regard to the allocation of medical resources, our study revealed that the enhancement of nursing care tends to increase the provision of long-term hospitalization care, which in turn, increases the demand for acute care. This trend supports Innami’s [40] observation that older adults tend to be accepted for long-term care hospital beds as a substitute for nursing homes, and for general hospital beds as an alternative to long-term care hospital beds. Additionally, our study suggests that physicians and nurses tend to be lower in number for long-term hospitalization beds and clinic beds, which is particularly pronounced in typical urban areas or remote regions. In urban areas, physicians and nurses tend to be concentrated in acute care because of the abundance of universities with a medical faculty providing advanced acute care. Conversely, in remote regions, the reduced demand for care discourages the establishment of nursing homes, resulting in an uneven distribution of care across different areas. Therefore, supplementing the medical and care supply through long-term hospitalization and clinic beds is essential to address the uneven distribution of care.

Second, concerning the allocation of care resources, our study suggests that the MHLW may not be able to reduce the number of hospital beds as intended through the enhancement of nursing homes alone, especially in middle-density regions, except for in typical urban areas or remote regions. Therefore, constructing numerous nursing homes in such regions may not be highly effective for reducing hospital beds. Moreover, from the urban planning perspective, middle-density regionality areas are of concern with regard to urban sprawl. Consequently, the location of nursing homes in such areas should be monitored carefully to ensure the efficient provision of care, reduction of hospital beds, and prevention of overdevelopment.

## Data Availability

The datasets generated and analyzed during the current study are available from the corresponding author on reasonable request.

## Abbreviations

CFI: Comparative fit index
df: Degrees of freedom
EFA: Exploratory factor analysis
MHLW: Ministry of Health, Labour and Welfare
PCA: Principal component analysis
PC: Principal component
RMSEA: Root mean square error of approximation
SEM: Structural equation modeling
SMA: Secondary medical area
SRMR: Standardized root mean square residual
TLI: Tucker–Lewis index

## References

[1] Cabinet Office: Annual Report on the Ageing Society FY. 2022. 2022. https://www8.cao.go.jp/kourei/whitepaper/w-2022/html/zenbun/index.html . Accessed 18 Feb 2023.

[2] Muto M (2019). Japanese hospital beds and regional medical delivery concept. J Int Univ Health Welf.

[3] Ministry of Health, Labour and Welfare. Hospital Report. n.d. https://www.mhlw.go.jp/toukei/list/80-1.html. Accessed 24 Oct 2023.

[4] Ministry of Health, Labour and Welfare. About the Regional Medical Vision. 2020. https://www.mhlw.go.jp/content/10800000/000686050.pdf. Accessed 18 Feb 2023.

[5] Ministry of Health, Labour and Welfare. Status of Secondary Medical Areas. 2014. https://www.mhlw.go.jp/file/05-Shingikai-10801000-Iseikyoku-Soumuka/0000058300.pdf. Accessed 18 Feb 2023.

[6] Ministry. of Health, Labour and Welfare. Annual Health, Labour and Welfare Report 2018 Whole Edition. 2018. https://www.mhlw.go.jp/wp/hakusyo/kousei/18/dl/all.pdf. Accessed 18 Feb 2023.

[7] Ministry of Health, Labour and Welfare: Community-based Integrated Care Systems. n.d. https://www.mhlw.go.jp/stf/seisakunitsuite/bunya/hukushi_kaigo/kaigo_koureisha/chiiki-houkatsu/. Accessed 18 Feb 2023.

[8] Ministry of Health, Labour and Welfare. About the medical care plan. 2017. https://www.mhlw.go.jp/file/06-Seisakujouhou-10800000-Iseikyoku/0000159901.pdf. Accessed 18 Feb 2023.

[9] Boniol M, McCarthy C, Lawani D, Guillot G, McIsaac M, Diallo K (2022). Inequal distribution of nursing personnel: a subnational analysis of the distribution of nurses across 58 countries. Hum Resour Health.

[10] Yuan Y (2021). Public satisfaction with health care system in 30 countries: the effects of individual characteristics and social contexts. Health Policy.

[11] Matsumoto M, Inoue K, Bowman R, Noguchi S, Toyokawa S, Kajii E (2010). Geographical distributions of physicians in Japan and US: impact of healthcare system on physician dispersal pattern. Health Policy.

[12] Matsumoto M (2011). Geographic distribution of physicians: an international comparison. Iryo Shakai.

[13] Zhang B, He S, Chen X, Jiang L (2022). Determining the spatial distribution of nursing homes in China: a spatial heterogeneity analysis. J Hous Built Environ.

[14] Gu Z, Luo X, Chen Y, Liu X, Xiao C, Liang Y (2022). Density, diversity, and design: evaluating the equity of the elderly communities in three measures of the built environment. Land.

[15] Hara K, Otsubo T, Kunisawa S, Imanaka Y (2017). Examining sufficiency and equity in the geographic distribution of physicians in Japan: a longitudinal study. BMJ Open.

[16] Seo Y, Takikawa T (2022). Regional variation in national healthcare expenditure and health system performance in central cities and suburbs in Japan. Healthcare.

[17] Takizawa T, Kasa T (2021). Challenges and prospects of the Aomori regional medical care vision from the perspective of a medical social worker at a hospital with convalescent beds. Aomori J Health Welf.

[18] Miyazawa S (2021). Regional disparity of medical supply and demand in Japan as of 2025: analysis on the regional health vision number of estimation of necessary beds. Japanese J Reg Policy Stud.

[19] Ikeda T, Tsuboya T (2021). Place of death and density of homecare resources: a nationwide study in Japan. Annals Geriatric Med Res.

[20] Jin X, Mori T, Sato M, Watanabe T, Noguchi H, Tamiya N (2020). Individual and regional determinants of long-term care expenditure in Japan: evidence from national long-term care claims. Eur J Pub Health.

[21] Nishino T (2017). Quantitative properties of the macro supply and demand structure for care facilities for elderly in Japan. Int J Environ Res Public Health.

[22] Ishikawa M, Takahashi T (2013). Quantitative analysis of Japanese medical service level of each medical region. J Japanese Assoc Health Care Administrators.

[23] Kusunoki T, Yoshikawa T, Sanuki R (2023). Analysis of the distribution structure of the number of hospital beds and the capacity of long-term care insurance facilities from the viewpoint of regional characteristics: using structural equation modeling for secondary medical areas throughout Japan in 2015. J Archit Plann AIJ.

[24] Ministry of Health, Labour and Welfare. Basic Policy for Ensuring Comprehensive Medical and Care Service in the Region Policy for Comprehensive Assurance of Medical and Care. 2016. https://www.mhlw.go.jp/file/05-Shingikai-12401000-Hokenkyoku-Soumuka/0000146722.pdf. Accessed 18 Feb 2023.

[25] Statistics Bureau. Population Census. n.d. https://www.stat.go.jp/english/data/kokusei/index.html. Accessed 17 Nov 2022.

[26] Miyake T, Satoh E, Mitsuhashi N, Kumakawa T (2016). A study on disparity in access to medical facilities from the viewpoint of regional characteristics. J Archit Plann AIJ.

[27] Statistics Bureau. Report on Internal Migration in Japan. n.d. https://www.stat.go.jp/english/data/idou/index.html. Accessed 17 Nov 2022.

[28] Statistics Bureau. Economic Census. n.d. https://www.stat.go.jp/english/data/e-census.html. Accessed 17 Nov 2022.

[29] Ministry of Internal Affairs and Communications. Settlement of Accounts by Municipality. n.d. https://www.soumu.go.jp/iken/kessan_jokyo_2.html. Accessed 17 Nov 2022.

[30] Ministry of Land, Infrastructure, Transport and Tourism. National Land Numerical Data: Land Use Tertiary Mesh Data. n.d. https://nlftp.mlit.go.jp/ksj/gml/datalist/KsjTmplt-L03-a-v3_1.html. Accessed 17 Nov 2022.

[31] National Federation of Depopulated Areas. Data Bank of Depopulated Areas. n.d. https://www.kaso-net.or.jp/publics/index/19/. Accessed 17 Nov 2022.

[32] Revelle W (2022). Psych: procedures for personality and psychological research.

[33] R Core Team. R: A language and environment for statistical computing. Vienna: R Foundation for Statistical Computing. 2022. https://www.R-project.org/.

[34] Mnistry of Health, Labour and Welfare. Survey of Medical Institutions. n.d. https://www.mhlw.go.jp/toukei/list/79-1.html. Accessed 30 Nov 2022.

[35] Japan Medical Association Research Institute. Current State of Local Medical Delivery Systems - Data Collection by Prefecture and Secondary Medical Area (8th Edition., April 2020). 2020. https://www.jmari.med.or.jp/download/WP443/WP443.pdf. Accessed 30 Nov 2022.

[36] Ministry of Education, Culture, Sports, Science and Technology. List of universities with faculty of medicine (2019). 2019. https://www.mext.go.jp/component/a_menu/education/detail/__icsFiles/afieldfile/2019/08/30/1325992_001.pdf. Accessed 30 Nov 2022.

[37] Rosseel Y (2012). lavaan: an r package for structural equation modeling. Journal of Statistical Software..

[38] Schermelleh-Engel K, Moosbrugger H, Müller H (2003). Evaluating the fit of structural equation models: tests of significance and descriptive goodness-of-fit measures. Methods Psychol Res Online.

[39] Ministry of Health, Labour and Welfare. Guidelines for Regional Medical Visions. n.d. https://www.mhlw.go.jp/content/10800000/000711355.pdf. Accessed 18 Feb 2023.

[40] Innami I. A study of social hospitalization. 1st ed. Tokyo: TOYO KEIZAI INC; 2009.

[41] Suzuki W, Yasushi I, Yuda M, Morozumi R (2012). The distribution patterns of medical care and long-term care expenditures: estimations based on administrative data in Fukui prefecture. Japanese J Health Econ Policy.

[42] Oguro K, Hirakata K, Policy Research Institute, Ministry of Finance, Japan (2017). A consideration of the location of nursing homes and the use of GIS geographic information system under the declining population and super-aging population: Niigata city as a case study. Financ Rev.

[43] Asano J, Matsushita K (2018). A study on locational characteristic of welfare facilities on care insurance in urbanization control area of local cities in the case of day care facilities and nursing homes in Toyohashi city. J City Plann Inst Japan.

[44] Nakazono M, Mishima S, Yamamoto S, Koh S (2019). Location pattern and use sphere of day care facilities for the elderly in Hagi area of Yamaguchi prefecture: arrangement planning theory of day care facilities in rural districts. J Archit Plann AIJ.

[45] Nakazono M, Mishima S, Yamamoto S, Koh S (2019). Establishment effect of day care facilities for the elderly by local governments in Suo Oshima town: site planning method of day care facilities for the elderly in island areas. J Archit Plann AIJ.

[46] Tokito M, Nishino T (2018). Simulation of optimizing capacity of facility for the elderly at a city where the senior population has decreased. AIJ J Technol Des.

[47] Ibuka Y, Matsuda Y, Shoji K, Ishigaki T (2020). Evaluation of regional variations in healthcare utilization. Japanese J Stat Data Sci.

[48] Jin X, Iwagami M, Sakata N, Mori T, Uda K, Tamiya N (2022). Regional variation in long-term care spending in Japan. BMC Public Health.

[49] Johnson EM. Physician-induced demand. Encyclopedia Health Econ. 2014;77–82. 10.1016/B978-0-12-375678-7.00805-1.

